# Factors Affecting Long-Term Compliance of CPAP Treatment—A Single Centre Experience

**DOI:** 10.3390/jcm11010139

**Published:** 2021-12-27

**Authors:** Agata Gabryelska, Marcin Sochal, Bartosz Wasik, Przemysław Szczepanowski, Piotr Białasiewicz

**Affiliations:** 1 Department of Sleep Medicine and Metabolic Disorders, Medical University of Lodz, 92-215 Lodz, Poland; agata.gabryelska@gmail.com (A.G.); sochalmar@gmail.com (M.S.); wasikbartosz92@gmail.com (B.W.); 2 Medical Simulations Center, Medical University of Lodz, 92-213 Lodz, Poland; przemyslaw.szczepanowski@umed.lodz.pl

**Keywords:** CPAP (continuous positive air pressure), PSG (polysomnography), OSA (obstructive sleep apnoea), adherence

## Abstract

Continuous positive airway pressure (CPAP) has been the standard treatment of obstructive sleep apnoea/hypopnoea syndrome (OSA) for almost four decades. Though usually effective, this treatment suffers from poor long-term compliance. Therefore, the aim of our one centre retrospective study was to assess factors responsible for treatment failure and long-term compliance. Four hundred subsequent patients diagnosed with OSA and qualified for CPAP treatment were chosen from our database and compliance data were obtained from medical charts. Many differing factors kept patients from starting CPAP or led to termination of treatment. Overall, almost half of patients ended treatment during the mean time of observation of 3.5 years. Survival analysis revealed that 25% of patients failed at a median time of 38.2 months. From several demographic and clinical covariates in Cox’s hazard model, only the presence of a mild OSA, i.e., AHI (apnoea/hypopnoea index) below 15/h was a factor strongly associated with long term CPAP failure. The compliance results of our study are in line with numerous studies addressing this issue. Contrary to them, some demographic or clinical variables that we used in our survival model were not related to CPAP adherence.

## 1. Introduction

Obstructive sleep apnoea/hypopnoea syndrome (OSA) is a prevalent disorder with increasing medical, economic, and social burden. As in any other medical condition, diagnosis and implementation of proper treatment are essential. The third, often neglected part of paramount importance, is securing adherence to therapy.

Continuous positive airways pressure (CPAP) has been considered the treatment of choice for moderate and severe OSA for almost 40 years [1]. It is highly effective and usually well tolerated. Nevertheless, there are a group of patients who either fail to start the treatment in spite of clear indications or are not compliant in the long term. Long-term compliance data are usually limited, as many patients are lost to follow up or attend control visits irregularly. This is probably why differing data regarding long term compliance have been published. In one early study, compliance after 5 years was only 68% [2]. Pepin et al., have recently published results of a nationwide study on CPAP adherence and found that termination rate after 3 years of treatment was almost 50% [3]. Other studies based on data from a large cloud database reported 90 days adherence at the level of only 75% [4]. Interestingly, despite technical progress in masks design and materials, and also modes of positive airway pressure delivery, it seems that non-compliance rate has remained as high as 34% and has not improved over the last 20 years [5].

There are numerous factors that may affect adherence to CPAP treatment. For instance, several demographic and clinical variables, e.g., age, sex, BMI (body mass index), race, apnoea-hypopnoea index (AHI), Epworth Sleepiness Scale (ESS) score and the presence of comorbidities were shown to have an effect on compliance [2,3,6,7]. Conversely, in the SAVE (The sleep apnea cardiovascular endpoints) study only compliance at 1 month and the presence of side effects of CPAP therapy were predictors of its use at 12 months [8]. Moreover, some interventions may favourably influence CPAP adherence. One of the meta-analyses reported that surveillance via telemedicine was valuable in improving compliance [9]. Likewise, a small study revealed that a smartphone application could improve short term CPAP adherence [10]. Another study pointed out that a chance to acclimate to CPAP before a titration night improved short term CPAP usage [11]. To sum up, long-term compliance despite technical improvement in CPAP devices and masks remains unsatisfactory and there is no consensus which demographic or clinical factors lead to treatment failure.

Therefore, the aim of our study was to assess the frequency of patients that start CPAP treatment and their long-term compliance, and furthermore, to reveal factors responsible for treatment failure in order to contribute to this research area.

## 2. Materials and Methods

### 2.1. Patients

In a retrospective study, a group of OSA patients were referred for CPAP treatment from the beginning of 2012 to the end of 2015. All OSA diagnoses were based on an in-patient, attended standard diagnostic polysomnography (PSG). Four hundred subsequent patients diagnosed with OSA had been qualified to a CPAP titration night under PSG supervision. The majority of them had AHI ≥ 15 (N = 346). The remaining 54 patients had AHI < 15 calculated for total sleep time (TST). Most of them presented with positional OSA, i.e., their ratio of AHI in supine position to AHI in the lateral position was ≥2 (N = 51) with supine AHI ≥ 15 and the lateral one < 15 (N = 42). The remaining three subjects slept only in the lateral position and consequently their supine AHI was unknown. Those with overall AHI < 15 qualified for CPAP trial presented with typical symptoms (e.g., daily somnolence or unrefreshing sleep) or cardio-vascular comorbidities.

### 2.2. Schedule of the Study Visits

All referred patients with presumptive OSA diagnosis had a first ambulatory visit at the centre in order to assess the risk of OSA by obtaining information on demographics, medical history regarding typical OSA symptoms (i.e., daily somnolence, unrefreshing sleep, witnessed apnoea’s), comorbidities, and conducting a general physical examination. One of the tools to assess daily somnolence routinely used at the visit was Epworth Sleepiness Scale [12]. Next, those with probable OSA diagnosis were qualified to diagnostic, supervised PSG at the centre. After PSG, they attended a second ambulatory visit in order to discuss current diagnosis and treatment options. Patients with moderate or severe OSA and with mild OSA (symptomatic or with cardio-vascular comorbidities) were qualified for CPAP titration night under PSG surveillance. At this visit, they also had an opportunity to try different nasal and full-face masks in order to choose the most comfortable/suitable one. Moreover, they had a chance to familiarize with CPAP breathing through the mask and to find the comfortable ramp pressure. Usually, within one week after their visit, they were admitted for a CPAP titration trial night at the centre. Afterwards, they attended the third ambulatory visit to discuss efficacy of CPAP treatment and its tolerance. In case of effective and well tolerated CPAP titration, patients were provided with a reimbursement form for a national health insurer and instructed on the choice and costs of different CPAP devices available on the market at that time. They were also asked to come to the first control visit within 3 months from the start of ambulatory CPAP therapy. After the first control visit, unless there were no treatment problems to solve, they were asked to schedule control visits twice a year.

### 2.3. Diagnostic Polysomnography

A standard night polysomnography was performed to obtain OSA diagnosis and was also undertaken during CPAP titration night. Patients were admitted to the sleep lab at 21:00 h (±0.5 h) and underwent physical examination (measurement of body mass, height, heart rate and blood pressure). A standard nocturnal polysomnography was performed by recording the following channels: electroencephalography (C4/A1, C3/A2), chin muscles and anterior tibialis electromyography, electrooculography, measurements of oro-nasal air flow (a thermistor gauge), snoring, body position, respiratory movements of chest and abdomen (piezoelectric gauges), unipolar electrocardiogram and haemoglobin oxygen saturation (SaO_2_) (Sleep Lab, Jaeger-Viasys, Hoechberg, Germany). Sleep stages were scored according to the criteria based on the 30 s epoch standard [13]. Apnoea was attained with the reduction of air flow to less than 10% of the baseline for at least 10 s. Hypopnoea was defined as at least 30% reduction of air flow for at least 10 s, accompanied by a 4% or greater decrease in SaO_2_ or an arousal. EEG arousals were scored according to AASM (American Academy of Sleep Medicine) guidelines [14,15]. All polysomnograms were manually scored by the qualified physicians at the centre.

### 2.4. CPAP Titration under PSG Surveillance

CPAP with humidifier and an A-flex (RemStar Auto, Philips Respironics, Murrysville, PA, USA) was set in automatic titration mode with ramp pressure from 4 to 6 and maximal one of 20 mbar and a ramp time usually from 20 to 30 min. Effective CPAP treatment pressure was defined as the one encompassing 90% of pressures delivered by auto-CPAP. Efficacy of CPAP treatment was defined as: at least 50% reduction of AHI to a level below 15/h for total sleep time (TST) or in case of positional OSA, for sleep in the supine sleeping position.

### 2.5. Compliance Assessment of Long-Term CPAP Treatment

To verify CPAP compliance and duration of treatment, patients’ medical charts were reviewed in 2019. From the whole group of 400 patients (315 males) we managed to collect data regarding CPAP usage from at least one control visit for 309 subjects (248 males) at a mean time of 43 ± 10 months from the titration night. They were ascribed to one of the following groups: CPAP titration failure, lost to follow up (no visit recorded after CPAP titration night), primary failures (those who did not start CPAP treatment despite successful titration), secondary failures (patients that began ambulatory CPAP therapy but terminated it) and still on treatment (patients who at the last recorded control visit reported current CPAP usage). Routinely, at the control visits, patients were asked about regularity of CPAP use. A regular user was defined as someone who sleeps with CPAP at least four hours per night and at least 5 nights per week.

### 2.6. Data Management and Statistical Analysis

Statistica 13.3 (StatSoft, Krakow, Poland, 2017) was used for the purpose of statistical analysis. All variables were formally tested for normal distribution with Shapiro–Wilk test. Data with normal distribution were presented as a mean ± SD; for non-normal distribution, a median and upper and lower quartile were shown. Categorical data were presented as frequencies. Statistics included *t*-test and Mann U Whitney test with continuity correction to test differences between groups with normal and non-normal distribution, respectively. Differences in frequencies were compared using Chi^2^ test with Yates’ correction. To assess the compliance of CPAP treatment, we constructed a Kaplan–Meier survival analysis supported by the Cox’s hazard model. In a Kaplan–Meier survival analysis, we compared the effect of binary variables: sex, AHI < 15 vs. AHI ≥ 15 and positional vs. non-positional OSA status on long-term CPAP compliance using Wilcoxon by Gehan test. Moreover, we created the Cox’s hazard model using the following covariates: continuous (age, BMI, ESS score) and categorical ones (sex, AHI ≥ 15 vs. < 15, positional vs. non-positional OSA), while the CPAP survival time was a dependent variable. A *p*-value below 0.05 was considered significant.

## 3. Results

### 3.1. Study Population

**Figure 1:**
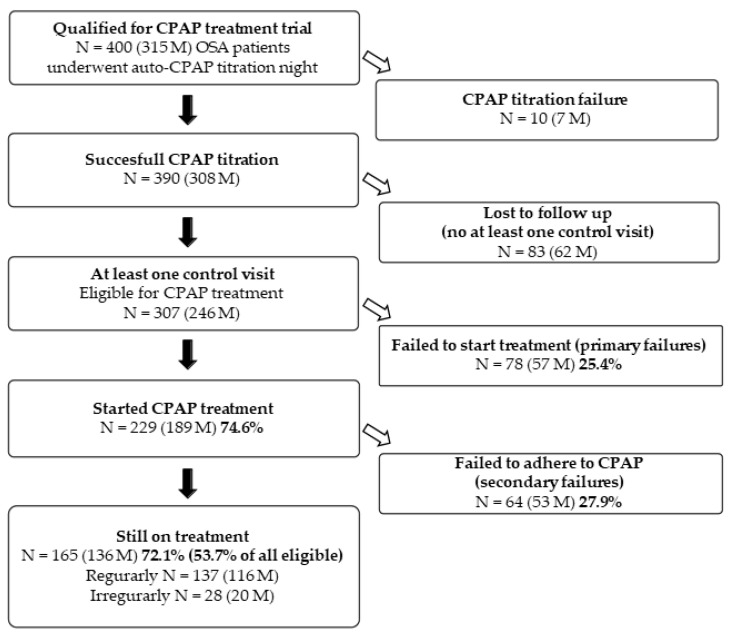
Flow chart of the study. CPAP—continuous positive airway pressure, M—male, N—number, OSA—obstructive sleep apnoea/hypopnoea syndrome.

From the initial 400 patients (315 males) who underwent auto-titration night, only 10 were failures: four patients (3 males) did not tolerate CPAP, CPAP was not effective in 5 patients (3 males) and one male had a predominance of central apnoea’s. Thus, the first night of the auto-CPAP titration trial was tolerated and effective in 97.5% patients. From the group of 390 patients who underwent successful CPAP titration, we found information regarding CPAP usage on 307 of them (246 males) at a mean time of 43 ± 10 months from the titration night, which means that 83 (21.3%) patients were lost to follow-up, i.e., they did not attend any control visit at the centre and it was not possible to confirm whether they started treatment. Only 229 (74.6%, 189 males) out of 307 eligible for treatment started CPAP; the remaining 78 (25.4%, 57 males) did not, and thus were considered the primary failures. At the time of the last recorded control visit, 165 (72.1%, 136 males) were still under treatment. Regular usage (on average at least 5 nights weekly of at least 4 h per night) was reported by 137 patients (83.0%, 116 males), while 28 (17%, 20 males) reported irregular use. A secondary failure (i.e., discontinuation after beginning of ambulatory CPAP treatment) was reported by 64 patients (29.7%, 53 males). To summarise, due to primary and secondary failures, at the time of the last recorded visit, 53.7% of all eligible patients were still under treatment and 44.6% reported treatment on regular basis. The flow chart of the study is presented in Figure 1.

### 3.2. Characteristic of Patients

OSA patients eligible for CPAP treatment were a heterogenous group and their demographic and clinical characteristics are summarized in Table 1. As expected, the majority (80%) were males; the females were older and had marginally lower AHI on CPAP, 90% CPAP pressure and ESS score.

### 3.3. Causes of CPAP Titration, Primary and Secondary Failures

The reasons given by patients that were qualified as titration failures, declined to start CPAP treatment (primary failures) or ended the treatment (secondary failures) are summarized in Table 2. The main reasons for primary failure were the high cost of the CPAP device, the preference for other treatment, or CPAP intolerance. Likewise, in the majority of cases, secondary failure was brought about by CPAP intolerance or the preference for other treatment.

### 3.4. Characteristic of Patients That Initiated CPAP, Primary Failures, Patients on Treatment and Secondary Failures

A comparison of the demographic and clinical data of primary and secondary failures, and patients that initiated CPAP or were still on treatment, are summarised in Table 3 and Table 4. In general, all CPAP failures (primary and secondary combined) were related to lower by 25% median AHI and 10% higher frequency of mild OSA; in case of primary failures marginally lower than mean age.

### 3.5. Analysis of Long-Term Compliance

**Figure 2:**
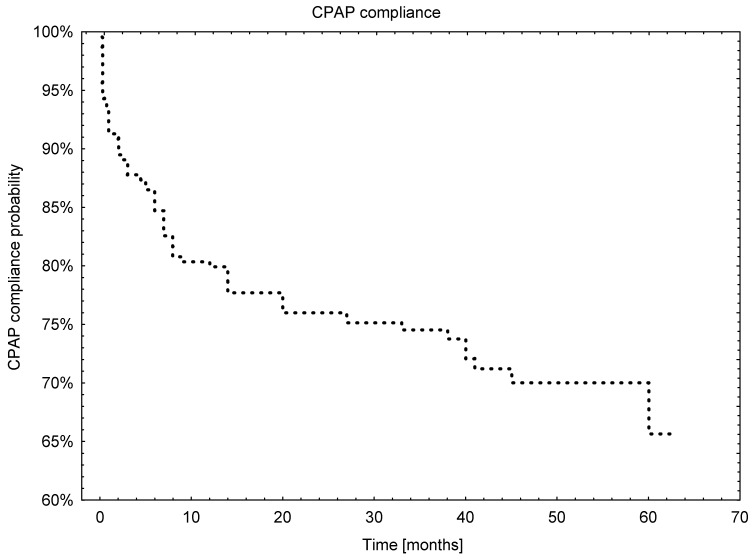
The overall Kaplan–Meier curve for CPAP usage.

From the 229 patients who started CPAP treatment, more than 50% were still on treatment at the time of their last recorded visit and 25% of patients who started CPAP treatment failed at a median time of 38.2 months. Nevertheless, from the Kaplan–Meier curve it can be inferred that the 20% drop in compliance occurred in the first 10 months, while during the subsequent 50 months the CPAP adherence dropped by 25%. The overall survival analysis is presented in Figure 2.

**Figure 3:**
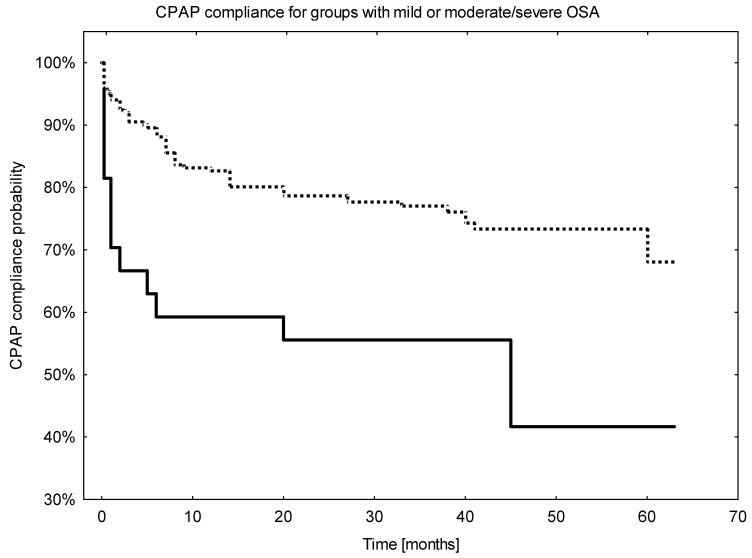
The Kaplan–Meier curves for CPAP usage calculated for groups of patients with moderate or severe OSA (AHI ≥ 15) and mild OSA (AHI < 15). solid line—patients with AHI < 15, dotted line—patients with AHI ≥ 15.

Sex or the presence of positional OSA did not influence CPAP compliance, *p* = 0.85 and *p* = 0.48, respectively. Otherwise, at AHI cut-off of 15 (mild vs. moderate or severe disease) compliance was worse for patients with AHI below this threshold (43.2%) vs. those with AHI ≥ 15 (67.9%), *p* = 0.003. For patients with AHI < 15 the drop in compliance was 40% in less than 10 months vs. 15% for the group with AHI ≥15, Figure 3.

In the last step we performed an analysis of proportional regression of Cox’s hazard, which is summarized in Table 5. This model yielded similar results to the comparisons of survival curves with only a binary variable based on AHI ≥ 15 or < 15 as a covariate with effect on CPAP compliance.

## 4. Discussion

CPAP, which is considered a treatment of choice for patients suffering from moderate to severe OSA is not easily accepted. Many diverse factors may cumulate leading to patients’ failure to initiate CPAP or non-compliance. Our retrospective study tried to assess long term compliance at a mean observation time of 3.5 years and recognize some of the factors responsible for non-adherence in hope to improve long-term therapy results. It appears that there are two important steps in case of ensuring CPAP compliance. The first one is to convince patients to begin treatment after a successful CPAP titration night in order to avoid a primary failure. The second one is to see to regular and long-term use, i.e., to avoid secondary failure. We found a substantial decline of CPAP use within our cohort at both steps, resulting in long term adherence at the frequency of 53.7% at the median time of 42 months. Additionally, only 44.6% of all eligible for treatment used CPAP on regular basis. Our results are concordant with the ones reported by several research groups over the last 30 years. In one early study, the CPAP compliance dropped to 80% after 3 months, which is comparable to our finding of 20% fall during the first 10 months [16]. A large study evaluating data from the large cloud database showed that only 75% of patients adhered to CPAP after 90 days [4]. Other group reported 50% CPAP failure after 3 years, which is consistent with our results [3]. Moreover, we found that the only variable that had an influence on long-term compliance was AHI level of 15, which is an arbitrary threshold between mild and moderate/severe OSA. Our study did not confirm the effect of some other factors/clinical variables on CPAP adherence recognized by other authors such as ESS, BMI, age, sex [2,3,17,18]. Interestingly, the same level of AHI was a factor of poor compliance in one of the early studies on the subject [19]. A study designed to evaluate factors of adherence to CPAP did not find the association with demographic or polysomnographic variables, which is partly in agreement with our negative results in respect of sex, BMI and age [20].

The reasons for primary failure seems to be diversified. The issue is quite complex with different factors playing a major role in different countries due to local regulations. One of them is economic, i.e., the level of reimbursement and, in effect, the final cost of CPAP. In our cohort, 24 patients (28% of all primary failures) reported that too high cost of acquisition was the reason for failure. Nevertheless, it is worth mentioning that it might have been biased, as the matter is delicate and, in effect, it is plausible that some patients did not want to admit this fact. In Poland, at that time, basic CPAP was reimbursed at the level of 70% leaving ca. 600 PLN (polish zlotys, ca. 170 USD) to be paid by the patient. Considering an average salary in the range of 3000–3500 PLN or an average pension in the range of 1500–2000 PLN per month, it was a considerable expenditure and, in effect, an economic barrier. The second largest group of primary failures comprised patients that decided to try another treatment: a positional one in case of positional OSA, diet, bariatric or uvulo-palatal surgery. Some of them, despite a successful first CPAP titration night, did not tolerate it from the beginning of ambulatory treatment.

It was our standard procedure that all study patients were given the chance to accommodate to CPAP treatment only for one night, which may be another reason for primary or secondary failure. One of the solutions to this problem may be a system that allows patients a longer accommodation time. In our centre, we started a telemedicine program of CPAP ambulatory titration that plausibly, in the long term, can give a better treatment survival time, as was reported by some researchers [10]. In this way patients can try a few different mask and experience ambulatory treatment for at least 7 nights. However, this hypothesis awaits evaluation.

In case of chronic diseases like OSA patients are more inclined and compliant to treatment if they experience the burden of symptoms and a relief upon therapy. Indeed, in some previously published reports daily hypersomnolence usually measured by ESS was a predictor of compliance [2,17,18]. One can expect the more severe disease, the higher probability to be symptomatic. Nevertheless, there is a subgroup of patients, even with severe OSA, that do not present with typical symptoms of substantial intensity. In such pauci-symptomatic patients even effective treatment may not be recognized as beneficial and, in effect, can lead to failure. In our model, ESS did not have an effect on compliance. This may be related to the fact reported by some authors that ESS score poorly correlated with OSA severity [21]. Similarly, to our results, it was not predictive of CPAP compliance in one of the studies [22]. Another probable factor of compliance may be more strict surveillance of therapy by the control visits, which, when frequent enough, can lead to recognition of ongoing treatment problems, solving them and thereby encouraging the compliance.

It seems that at a mean time of 3.5 years of follow up, only around 50% of OSA patients that are still using CPAP is a low value. It is similar to the results reported by many groups from the onset of using CPAP on a large scale for OSA treatment in the late 1980s. Additionally, in our opinion, it should be pointed out that this has not been significantly improved by the progress that has occurred in the field of CPAP devices and mask design and materials [5]. To illustrate this notion, a promising development of A-flex mode (which is a decrease in PAP pressure to ease exhalation) did not substantially affect adherence to treatment [23]. Therefore, small steps and improvements need to be taken to increase this outcome in the long term. Some of those factors were recognized in this study and may help to initiate improvements, but it seems that the most important aspect is human involvement, with more effort on the side of health care providers to supervise the CPAP treatment and find some systemic solutions to it.

There are some serious limitations to our study. It was conducted only in one centre and only on a moderate number of Caucasian patients. Moreover, a substantial fraction of patients were lost to follow up, thus their CPAP treatment status could not have been evaluated. The timing of control visits was irregular because it was a retrospective study and patients planned visits at their discretion. We did not obtain objective data on CPAP usage as the majority of devices used by our patients at that time did not store compliance data. Furthermore, for the purpose of the analysis, we chose a limited number of variables, omitting some of plausible importance, e.g., smoking status or comorbidities. Similarly, we did not analyse data on type of the mask (nasal vs. oro-nasal) or CPAP devices, which also may be of potential importance. Nevertheless, we believe, that this study may help to recognize factors responsible for CPAP non-adherence and in this way will contribute to ameliorating the persistent issue of CPAP non-compliance.

## Figures and Tables

**Table 1:** Demographic and clinical characteristic of patients.

	All Subjects N = 307	Males N = 246	Females N = 61	*p*
Age [years]	56.9 ± 10.8	55.9 ± 11.3	61.0 ± 7.3	<0.001
BMI [kg/m^2^]	33.2 ± 5.5	33.2 ± 5.5	33.0 ± 6.1	0.790
AHI	35.0, 21.0–56.4	37.8, 21.0–58.3	29.9, 21.2–46.0	0.068
AHI ≥ 15	40.6, 25.3–60.0 N = 265	43.0, 26.0–61.0 N = 214	33.0, 23.0–53.4 N = 51	0.081
AHI < 15	10.9, 8.2–13.0 N = 42	11.0, 8.1–13.1 N = 32	9.1, 8.3–13.0 N = 10	0.525
AHI CPAP	4.0, 2.0–11.0	5.0, 2.0–12.0	3.0, 1.0–6.0	0.005
90% CPAP pressure [mbar]	11.3 ± 2.3	11.5 ± 2.4	10.7 ± 2.4	0.033
ESS	9.5, 6.0–13.0	10.0, 6.0–13.0	8.0, 5.0–12.0	0.047

AHI—apnoea/hypopnoea index, BMI—body mass index, CPAP—continuous positive airway pressure, ESS—Epworth Sleepiness Scale score, N—number. All data are presented as a mean ± SD or median, lower–upper quartiles.

**Table 2:** Causes of CPAP titration, primary and secondary failures.

Causes of CPAP Failure	Titration Failure N = 10 out of 400	Primary Failure N = 78 out of 307	Secondary Failure N = 64 out of 229
CPAP titration pressure not effective	5 (3 M)	NA	NA
Predominant central apnoea	1 M	NA	NA
CPAP was not tolerated	4 (3 M)	15 (9 M)	31 (23 M)
CPAP was too expensive	NA	24 (19 M)	NA
Preferred other treatment *	NA	18 (16 M)	11 (10 M)
Afraid of CPAP treatment	-	4 (2 M)	-
Treatment of comorbidities was more important	NA	3 (1 M)	-
Did not feel benefit	-	-	2 (1 M)
Spontaneous improvement	NA	NA	8 (7 M)
Other reasons or declined to answer	NA	14 (10 M)	12 M
Total N	10 (7 M)	78 (57 M)	64 (53 M)

* Other treatment: weight reduction (diet, bariatric surgery, palate surgery), positional treatment (i.e., avoiding sleep in supine position). CPAP—continuous positive airway pressure, NA—not applicable, M—males, N—number.

**Table 3:** Demographic and clinical characteristics of patients who initiated CPAP and primary failures.

	Initiated CPAP N = 229	Primary Failures N = 78	*p*
Age [years]	57.8 ± 10.2	54.2 ± 12.1	0.013
BMI [kg/m^2^]	33.2 ± 5.2	33.2 ± 6.2	0.960
Sex—M (%)	189 (82.5%)	57 (73.1%)	0.071
AHI	38.0, 22.0–58.3	28.9, 17.8–51.0	0.028
AHI CPAP	4.0, 2.0–11.0	4.0, 1.3–9.0	0.511
90% CPAP pressure [mbar]	11.4 ± 2.5	11.1 ± 2.3	0.307
AHI ≥ 15 [N]	202 (88.2%)	63 (80.9%)	0.099
Positional OSA	109/185 (59%) *	42/60 (70%) *	0.167
ESS	10.0, 7.0–13.0	8.0, 5.0–12.0	0.085

* The total number of cases is lower than for the whole group as some patients slept only in one position and their positional status was not to be assessed. AHI—apnoea/hypopnoea index, BMI—body mass index, CPAP—continuous positive airway pressure, ESS—Epworth Sleepiness Scale score, M—males, N—number, OSA—obstructive sleep apnoea/hypopnoea syndrome. All data are presented as a mean ± SD or median, lower–upper quartile or frequencies.

**Table 4:** Demographic and clinical characteristic of patients still on treatment, secondary failures, and all failures.

	Still on CPAP N = 165	Secondary Failures N = 64	*p*	All Failures N = 142	*p*
Age [years]	57.6 ± 10.0	58.2 ± 9.1	0.68	56.0 ± 11.0	0.208
BMI [kg/m^2^]	33.2 ± 5.6	33.1 ± 4.1	0.87	32.7 ± 5.3	0.932
Sex—M (%)	136 (82.4%)	53 (82.5%)	0.94	110 (77.5%)	0.346
AHI	40.6, 24.6–60.9	30.7, 16.5–52.1	0.017	30.4, 17.0–52.0	0.001
AHI CPAP	4.0, 2.0–11.0	4.0, 1.5–11.0	0.804	4.0, 1.3–10.0	0.826
90% CPAP pressure [mbar]	11.0, 10.0–13.0	11.0, 10.0–12.0	0.214	11.0, 10.0–12.0	0.079
AHI ≥ 15	151 (91.5%)	51 (79.7%)	0.024	114 (80.3%)	0.007
Positional OSA	74/129 (57%) *	35/56 (63%) *	0.624	77/116 (66%) *	0.188
ESS	10.0, 7.0–13.0	9.0, 6.0–13.0	0.706	9.0, 6.0–12.5	0.163

* The total number of cases is lower than for the whole group because some patients slept only in one position and their positional status was not possible to assess. AHI—apnoea/hypopnoea index, BMI—body mass index, CPAP—continuous positive airway pressure, ESS—Epworth Sleepiness Scale score, OSA—obstructive sleep apnea/hypopnea syndrome, M—males, N—number. All data are presented as a mean ± SD or median, lower–upper quartile or frequencies.

**Table 5:** Proportional regression of Cox’s hazard.

	Beta	*p*	HR (95% CI)
age	0.006	0.630	1.01 (0.98–1.03)
sex	−0.044	0.896	0.96 (0.49–1.85)
BMI	0.017	0.509	1.02 (0.97–1.07)
AHI ≥ 15	−0.947	0.005	0.39 (0.20–0.75)
Positional OSA	0.053	0.871	1.05 (0.56–2.00)
ESS	−0.009	0.741	0.99 (0.94–1.05)

AHI—apnoea/hypopnoea index, BMI—body mass index, CI—confidence interval, ESS—Epworth Sleepiness Scale score, HR—hazard ratio, OSA—obstructive sleep apnoea/hypopnoea syndrome.
